# Assessment of the Aging State for Transformer Oil-Barrier Insulation by Raman Spectroscopy and Optimized Support Vector Machine

**DOI:** 10.3390/s24237485

**Published:** 2024-11-24

**Authors:** Deliang Liu, Biao Lu, Wenping Wu, Wei Zhou, Wansu Liu, Yiye Sun, Shilong Wu, Guolong Shi, Leiming Yuan

**Affiliations:** 1 School of Information and Engineering, Suzhou University, Suzhou 234000, China; 2 College of Electrical and Electronic Engineering, Wenzhou University, Wenzhou 325035, China; 3 Suzhou Vocational and Technical College, Suzhou 234000, China; 4 School of Information and Computer, Anhui Agricultural University, Hefei 230036, China

**Keywords:** Raman spectroscopy, oil-barrier insulation, aging state assessment, baseline correction, support vector machine

## Abstract

Accurate assessment of the aging state of transformer oil-barrier insulation is crucial for ensuring the safe and reliable operation of power systems. This study presents the development of indoor accelerated thermal aging experiments to simulate the degradation of oil-immersed barrier insulation within transformers. A series of samples reflecting various aging states was obtained and categorized into six distinct groups. Raman spectroscopy analytical technology was employed to characterize the information indicative of different aging states of the oil-immersed barrier insulation. The raw Raman spectra were processed using asymmetric reweighted penalty least squares to correct baseline shifts, Savitzky–Golay (S-G) smoothing to eliminate fluctuation noise, and principal component analysis (PCA) to reduce data dimensionality by extracting principal components. A support vector machine (SVM) classifier was developed to discriminate between the Raman spectra and category labels. The SVM parameters were optimized using grid search, particle swarm optimization (PSO), and genetic algorithm (GA), yielding the optimal parameters (C and gamma). Notably, the grid search method demonstrated high efficiency in identifying the best combination of SVM parameters (***c*** and ***g***). Comparative analyses with varying numbers of principal components in SVM classifiers revealed that incorporating an optimal subset of PCA features achieved the highest classification accuracy of 94.44% for external validation samples, with only eight samples being misclassified into adjacent categories. This study offers technical support and a theoretical foundation for the effective assessment of the aging state of oil-barrier type insulation in transformers, contributing to the advancement of condition monitoring and maintenance strategies in power systems.

## 1. Introduction

Transformers are pivotal components of power transmission and distribution systems, with their condition being paramount to the overall safety and reliability of the power grid [1,2,3]. The risk of transformer failure escalates as the insulation system degrades [1]. The internal insulation system of oil–paper-insulated power equipment comprises a composite structure of insulating oil and paper, which, over time, is subject to degradation due to various stressors including electrical stress, heat, and environmental factors [1]. This degradation can diminish the electrical and mechanical properties of the insulation, thereby impacting the safe and stable operation of transformers. Precise identification of the aging stages of transformer oil-barrier insulation, particularly for units with extensive operational histories, is integral to forecasting transformer service life. Research into the monitoring of the aging state of oil-barrier insulation is not only of significant importance for predictive maintenance but also essential for ensuring the safe operation of power networks and facilitating efficient asset management [1,4].

To date, various methods have been employed to assess the aging of oil-barrier insulation. These include measurements of the degree of polymerization (DP) of the insulation paper [5], furfural content analysis, and dissolved gas analysis (DGA) of the insulating oil [6,7]. Despite their utility, each method presents distinct limitations [3]. Assessing the DP requires power supply disconnection and core sampling, which poses substantial challenges for field applications. Determining furfural concentration can be achieved through techniques such as high-performance liquid chromatography (HPLC) [6], ultraviolet (UV) spectrophotometry, and colorimetry [1,2]. However, these methods encounter several limitations. HPLC is hindered by complex procedures and demanding elution processes [6]. UV spectrophotometry can be compromised by instability and interference from other organic compounds present in transformer oil. Colorimetry, which depends on visual assessment, tends to offer lower accuracy and is prone to operator subjectivity. DGA typically utilizes gas chromatography (GC) [7], which faces its own set of challenges, including column performance degradation over time and the requirement for specialized laboratory operations.

The current literature suggests that predicting the aging condition of transformer paper insulation material necessitates a synthesis of direct and indirect methodologies. The direct approach concentrates on measuring the DP of cellulose through viscosity tests, which, despite offering precise DP assessment, is limited by its destructive nature that restricts on-site applicability. Indirect methods for assessing insulation aging include a spectrum of techniques such as partial discharge current (PDC) measurements [1], Raman spectroscopy [7,8], chemical indicator analysis [8], and dielectric frequency domain spectroscopy (FDS) [9]. The application of activation energy [10] and the Frequency-Dependent Dielectric Modulus (FDDM) [10] as indicators of insulation aging has proven effective in characterizing the deterioration of insulation within the FDS framework. Assessing the aging of dry-type insulation is relatively more accessible due to its minimal moisture migration, in contrast to oil-immersed cellulose insulation. However, the significant moisture content inherent in oil–paper insulation can confound FDS results, thereby affecting the accuracy of aging metrics [11]. Moreover, oil–paper insulation, featured with dynamic and nonlinear characteristics, presents a more intricate challenge for intuitive representation than dry-type insulation. In this context, an innovative diagnostic technique was proposed to counteract the influence of moisture fluctuations on FDS measurements, demonstrating a linear relationship between the DP of the insulating paper and the water distribution coefficient. Nonetheless, for practical field applications, environmental impacts on oil temperature must be taken into account [5,9].

Raman spectroscopy is a potent, non-destructive, and high-resolution analytical technique that elucidates the vibrational, rotational, and other low-frequency modes of materials [12,13,14]. It provides insights into molecular structures, crystal symmetries, and electron–phonon interactions, offering valuable information for a variety of materials [14]. Advances in laser technology and charge-coupled device (CCD) detection have propelled Raman spectroscopy to the forefront of qualitative and quantitative analyses in diverse fields, including food safety [15] and cosmetics [16]. Recently, its application with microfluidic chips in detecting furfural, acetone, and methanol in insulating oil are 0.43 mg/L, 1.04 mg/L, and 2.31 mg/L, respectively, and this provides a new approach for the online detection of oil–paper insulation equipment in theory [17]. Furthermore, Abbasi’s review of Raman spectroscopy’s potential in the early diagnosis of transformer faults gained more attention and success [10]. Transformer oil-barrier insulation, a critical component of transformers, is categorized based on insulating oil types into 10#, 25#, and 45#, with the 45# oil-barrier insulation being prevalent in low-temperature and ultra-high-voltage environments. Despite its significance, scant research has explored the use of Raman spectroscopy to assess the aging status of insulation composed of 45# insulating oil and paper. This study aims to investigate the Raman spectral characteristics of 45# oil-barrier insulation and to evaluate its potential in aging stage assessment for oil-immersed transformers.

In this study, we prepared a series of oil samples at various stages of aging using our custom-designed thermal aging apparatus. The Raman spectra of these samples, corresponding to different durations of thermal stress, were subsequently characterized using a Raman spectrometer. The raw spectral data underwent a series of preprocessing steps to enhance the quality and analytical utility of the spectra. Specifically, the asymmetric reweighted penalty least square (arPLS) algorithm was applied to correct baseline shifts. This was followed by Savitzky–Golay smoothing to eliminate fluctuation noise and an intensity threshold cutoff to remove background signals. Finally, principal component analysis (PCA) was employed to compress the data and extract the most informative principal components. A support vector machine (SVM) classifier was developed to discern relationships between the Raman spectrum and the aging categories of the samples. The optimization of the SVM’s parameters was performed using grid search, particle swarm optimization (PSO), and genetic algorithm (GA), each meticulously tuned to identify the optimal combination of parameters (C and gamma). This approach was essential for enhancing the classifier’s predictive accuracy and reliability. The primary objective of this research is to identify Raman spectral features that are indicative of the aging process in transformer oil-barrier insulation. Furthermore, we aim to evaluate the feasibility of utilizing Raman spectroscopy as a non-destructive tool for condition monitoring and predictive maintenance of transformers. By establishing a robust link between spectral data and insulation aging, this study contributes to the advancement of transformer diagnostics and the development of proactive maintenance strategies.

## 2. Materials and Methods

### 2.1. Samples Preparation of Oil-Barrier Insulation Aging Specimens

In this work, No. 45 mineral insulating oil and kraft insulating paper, with a thickness of 0.2 mm, were utilized. Prior to the experiment, the insulating oil underwent drying at 80 °C for 72 h, followed by cutting the insulating paper into pieces and drying them for 48 h. Once both components were dried, they were mixed in a mass ratio of 1:8 and bottled. The experimental temperature for heating the mixtures was deliberately set at 130 °C. This temperature is below the flash point of No. 45 insulating oil (135 °C), ensuring both the safety of the experimental procedure and the rapid thermal aging of the oil-barrier insulation samples. This approach avoids the drawbacks associated with lower temperatures, which would prolong the thermal aging process and hinder the desired acceleration. At the same time, it prevents the damage to the molecular structure of the insulating oil that could occur at excessively high temperatures, thereby maintaining the authenticity of the test results.

For this study, the accelerated thermal aging sample preparation equipment from China Dingxinyi Company was employed, specifically, the DXY-12H model (Power: 220 v/50 Hz, 1.2 kw) of digital display constant temperature oil bath. This equipment is widely used in the petroleum and chemical industries for applications such as distillation, drying, concentration of chemical substances, and the impregnation of chemicals or biological products. The oil bath consists of a main body, an oil pot, a pot cover, a temperature control system, and other components. Its heating element, a high-quality stainless steel tubular electric heating tube, is directly immersed in the oil bath for efficient heat exchange. This heating tube boasts high heating efficiency, minimal heat loss, and employs industrial-grade self-tuning PID technology for precise temperature control. Compared to traditional PID methods, this system offers reduced temperature overshoot, faster stabilization, and superior temperature accuracy. Additionally, the temperature controller (Ranged: RT + 5 °C~300 °C) provides features such as timing control, temperature error correction, and deviation alarm protection. The aging timespans for the oil-barrier ranged from 0 h to 336 h, and they are divided into six classes for subsequent qualitative analysis. A total of 64 oil samples were prepared in each class, and a total of 64 × 6 oil samples were made in this experiment.

### 2.2. Raman Spectroscopy Detection Device

**Figure 1:**
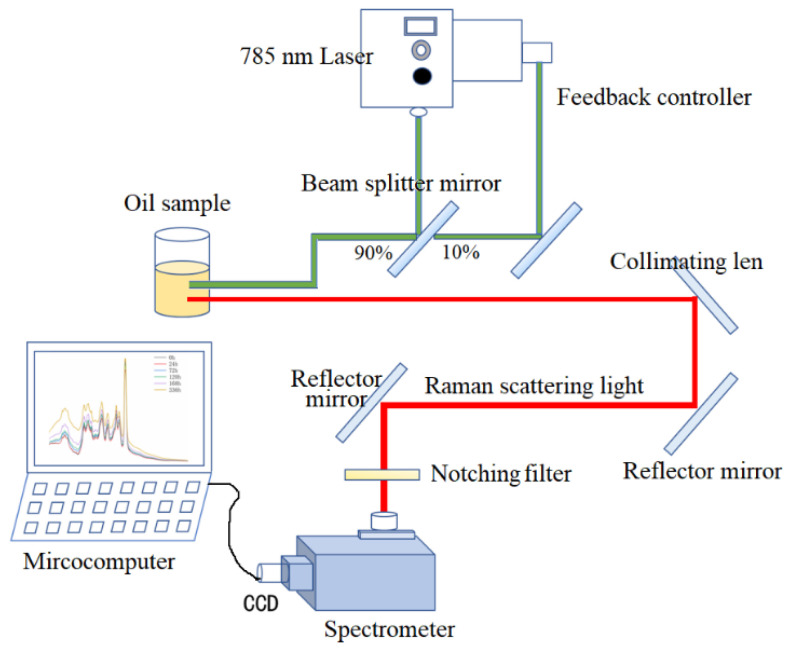
Diagram of oil sample testing device.

In this study, a specialized experimental setup was developed for the analysis of transformer oil-barrier using laser Raman spectroscopy (model ATR3110-785, produced by Xiamen AopuTiancheng Optoelectronic Co., Ltd. in Fujian, China), as its structure diagram depicted in Figure 1. The setup consists of a 785 nm laser, a fiber-optic probe, glass sample containers, a spectrometer, a microcomputer, and some ancillary components. Upon activation, the laser beam was emitted from the laser source and projected onto a 45° posed beam splitter mirror. The propagation direction of this short-pulse high-energy light beam was divided into two propagating directions. One beam (approximately 10% light energy) was transferred into the energy feedback controller, which regulates the pulse energy, and the other beam (close to 90% energy) was mainly converged by a convex lens (within the fiber-optic probe) and projected onto oil samples in a cuvette or glass bottle. Some laser light was absorbed, some was scattered (only a litter Roman-scattered light) and reflected, and some transmitted. Some scattered or reflected light beams were collected by light-collimating lens accessories, then filtered by a notch filter, and transferred to a spectrometer by an optics fiber. Inside the spectrometer, the scattered light undergoes photoelectric conversion, enabling the conversion of analog signals to digital signals. Subsequently, the digitized spectral signal is relayed to the microcomputer via a signal transmission line [18]. The microcomputer processes the spectral data through numerical calculations and analytical techniques, enabling the precise determination of the oil sample’s aging condition within the transformer.

Prior to the main experimentation, preliminary parameters comparisons were conducted to optimize the experimental parameter settings. Specifically, an integration time of 5 s, a laser power of 100 mW, and an average count of 4 were determined as the optimal settings. Averaging the spectra over 4 acquisitions helps to mitigate spectral noise, particularly Gaussian noise stemming from the spectrometer’s dark currents. These configurations were deliberately chosen to elevate the signal-to-noise ratio of the captured Raman spectra, mitigate interferences arising from stray light and fluorescence excitation, and ultimately yield more consistent Raman spectral readings from the oil samples.

### 2.3. Spectral Classification Algorithm

#### 2.3.1. Savitzky–Golay Smooth

The Savitzky–Golay (S-G) smoothing technique usually employs a local polynomial fitting approach in the sequential domain to serve as a signal filtering method [19,20]. A distinguishing attribute of this method is its ability to preserve the integrity of the signal’s form and breadth while effectively removing noise components. This smoothing process is typically used to “filter out” a noisy signal whose frequency span is broad.

#### 2.3.2. Asymmetric Reweighted Penalized Least Squares

To improve the effect of subsequent data processing, baseline correction is needed for the original spectral data after the above Savitzky–Golay smoothing [21,22]. In this study, asymmetric reweighted penalized least squares (arPLS) is employed for the baseline correction of Raman spectra [23]. The arPLS is a baseline correction algorithm proposed by Sung June Baek et al. of Chonnam University in Korea [24]. The weight updating process is expressed as follows:

(1) $$w_{i}=\left\{\begin{array}{c} \log i\;stic\left(y_{i}-z_{i},m_{d^{-}},\sigma_{d^{-}}\right)\;\;y_{i}\geq z_{i}\\ 1,\;\;\;\;\;\;\;\;\;y_{i}\leq z_{i} \end{array}\right.$$

(2) $$\log i\;stic(d,m,\sigma )=\frac{1}{\left\{1+\exp\left[\frac{2(d-(-m+2\sigma ))}{\sigma }\right]\right\}}$$

Among these parameters, ***y*** represents the original signal, ***z*** indicates the fitted signal, ***i*** stands for the ***i***-th point in a spectral signal, ***m*** is the mean value, and **δ** is the standard deviation of observations. When the difference value between the signal and the baseline is less than the estimated noise average, the logic function gives almost the same weight to the signal below or above the baseline, which avoids the baseline being underestimated in the non-peak region and overestimated in the peak region, and thus it ensures that the derived baseline is closer to the actual baseline. Otherwise, the weight ***w_i_*** is set to 1 to let the point value remain unchanged [23].

#### 2.3.3. Principal Component Analysis

The high-dimensional spectral data, with 2048 spectral points, presents challenges in the efficacy of classification algorithms. To mitigate these issues, principal component analysis (PCA) was employed for data dimensionality reduction [25]. PCA is a statistical procedure that efficiently transforms the original high-dimensional data into a lower-dimensional space, where the new variables, known as principal components (PCs), are constructed as linear combinations of the original variables. It was designed to capture the maximum variance within the data while retaining the essential characteristics of the original variables, thereby ensuring minimal information loss [26,27].

#### 2.3.4. Support Vector Machine

Support vector machine (SVM), introduced by Vapnik, is a supervised learning algorithm designed for pattern classification [28,29]. It functions based on the premise of identifying the most effective separating boundary, which aims to expand the separation between disparate groups within a dataset. SVM excels in binary classification by identifying the hyperplane that offers the greatest separation, which is achieved by minimizing the norm of the hyperplane’s normal vector. This approach ensures that the model is robust against overfitting and maintains generalization capabilities across various datasets.

SVM’s ability to handle low-dimensional spaces and a small number of samples makes it an ideal candidate for analyzing complex spectral datasets. The algorithm’s effectiveness in capturing the nuanced relationships within the data allows for precise classification, even when the decision boundaries are not linearly separable. Achieved by employing kernel functions, these tools transform the input data into an expanded dimensional realm where linear demarcation becomes viable. When confronted with multi-class classification problems, SVM employs strategies such as the One Against One (OAO) or One Against All (OAA) methods. The final classification decision is derived from the collective outcome of these binary decisions, often through a voting mechanism that reflects the majority consensus among the classifiers.

As it is known, there are two key parameters in the SVM algorithm that affect the performance of the classification model, namely, the penalty coefficient **c** and the kernel function parameter **g**. In this study, three methods can be found in the literature [30], namely, genetic algorithm (GA), grid search, and particle swarm optimization (PSO), which are used to optimize these two parameters in the SVM classification model. At the same time, cross-validation with 5 k-fold was used to avoid the overfitting or underfitting of the qualitative model [30].

## 3. Results and Discussion

### 3.1. Analysis of Spectral Profile

**Figure 2:**
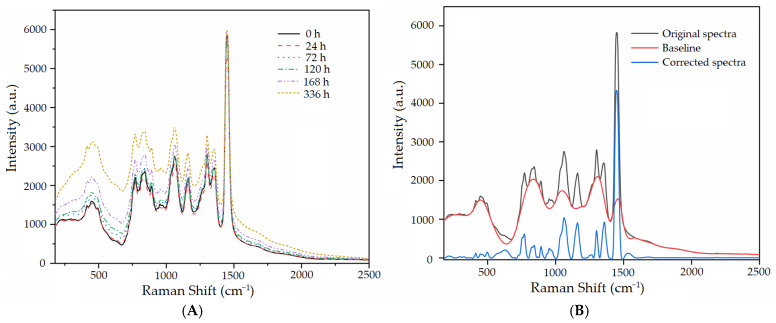
Raman spectra of the aging oil samples. (**A**) The average Raman spectra with various aging statuses. (**B**) Comparison of Raman spectra after baseline correction.

To better understand the tendency variations in Raman spectra of the oil-barrier insulation samples across different aging durations, Figure 2A illustrates the averaged spectrum for each aging timepoint derived from the raw spectral data. The aging timespan for the oil-barrier ranged from 0 h to 336 h, with a total of six designated intervals. Consequently, six curves are presented, each corresponding to the average spectra of the samples within these six categories. Examining the trends in spectral variations, it is observed that their spectral trends are largely similar, apart from differences in absorbance values. For instance, the average spectral curves labeled as 0 h and 24 h essentially overlap. As the aging duration increases, the intensity values of the Raman spectra also rise, particularly in the case of the sample with the longest aging time, which exhibits the highest Raman spectral intensity and deviates from the clustering range of the other five spectra. Among these spectra, the number of spectral peaks present is consistent, and the positions of the peaks also remain unaltered. Specifically, peak positions can be observed around 480 cm^−1^, 760 cm^−1^, 780 cm^−1^, 1060 cm^−1^, 1200 cm^−1^, 1310 cm^−1^, 1360 cm^−1^, and 1450 cm^−1^.

In Raman spectroscopy analysis, the fluorescence effect poses a common yet challenging issue. As most samples generate fluorescence under laser excitation, it leads to a baseline shift in the Raman spectrum, causing the spectral valleys to deviate from the zero axis. This situation is clearly illustrated in Figure 2A, where the original Raman spectrum is significantly affected by the fluorescence effect of the oil sample, resulting in notable spectral differences among various samples and obscuring the true variations in Raman peak intensities.

To address this problem, this study employs the arPLS method for baseline correction of the original Raman spectrum [23,24]. Taking the insulating oil sample with an aging time of 0 h as an example, the baseline-corrected spectrum is presented in Figure 2B. This figure displays the curves of the original spectrum, baseline spectrum, and baseline-corrected spectrum of samples with 0 h aging. It is evident that after baseline correction, the spectral valleys are effectively aligned close to the zero axis, thereby highlighting a greater number of Raman peaks. In particular, some minute Raman peaks that were previously concealed by the fluorescence effect are now clearly visible. In addition to baseline correction, it is common practice to perform smoothing processing on the corrected spectral data to further reduce noise interference. In spectral data exhibiting pulse peak characteristics, the Savitzky–Golay (S-G) smoothing method has been proven to be highly effective [19]. This approach not only emphasizes the local details of the spectrum but also filters out minor fluctuations caused by noise. Through such processing, we can obtain more accurate and reliable Raman spectral data, providing strong support for subsequent sample analysis and interpretation.

Analyzing from the viewpoint of components, the insulating paper is mainly composed of cellulose, which is a highly polymerized hydrocarbon composed of glucose monomers through glycosidic bonds. In the aging process, due to the influence of temperature, the glycosidic bonds connecting glucose monomers are broken to produce furfural and other aging substances dissolved in oil, and the longer the aging time, the more glycosidic bonds are broken [8,11]. Under the joint action of the aging substances in the oil and the insulating oil itself, the intensity of the Raman spectrum of the measured oil sample becomes stronger and stronger.

### 3.2. Division of Samples

According to the different timespan of the thermal aging process, oil samples aged with 0 h were assigned as class 1, oil samples aged with 24 h were assigned as class 2, and samples aged with other timespans of 72 h, 120 h, 168 h, and 336 h were labeled with class 3, 4, 5, and 6 labels, respectively, as shown in Table 1. Thus, there are six classes for the different aging timespans. Samples in each class were divided into two subsets with the ratio of 5:3 by the algorithm of SPXY. In each class, there were 64 different samples, of which 40 were divided into the calibration subset, and the remaining 24 in the same class were assigned as the prediction subset. Each Raman spectrum was obtained from one sample. Thus, finally a total of 40 × 6 oil samples in each subset were grouped into the calibration set, which was used to train the qualitative model. The remaining 24 × 6 oil samples were grouped into the prediction set, which was used to validate the feasibility of the above developed model.

### 3.3. Dimension Reduction by Threshold and PCA

Figure 2B demonstrates that following baseline correction, the matrix and fluorescence interferences from the samples are effectively mitigated in the Raman spectra. This enhancement allows for clearer visualization of the characteristic Raman peaks. In an ideal scenario, a complete Raman spectrum is composed of both the Raman characteristic peaks and the Raman background signal, which should theoretically approach zero after baseline correction [23,31]. However, in practical applications, it is challenging to completely eradicate the Raman background signal through algorithmic means. Nonetheless, its impact can be significantly minimized, thus improving the signal-to-noise ratio of the Raman spectral features. Consequently, the low-intensity regions of the Raman spectrum, particularly those beyond 1600 cm^−1^, can be considered as background signals and are candidates for exclusion to reduce interference and streamline subsequent analyses. We can establish an arbitrary threshold to disregard spectral points of lower intensity. By applying empirical evidence, we can eliminate approximately 40% of the spectral variables from initial attempts; specifically, when the spectral intensity threshold is set at 8.2, most of the spectral data beyond 1600 cm^−1^ can be disregarded, along with a small portion of the spectral region prior to 400 cm^−1^, and the valleys in other spectral regions.

**Figure 3:**
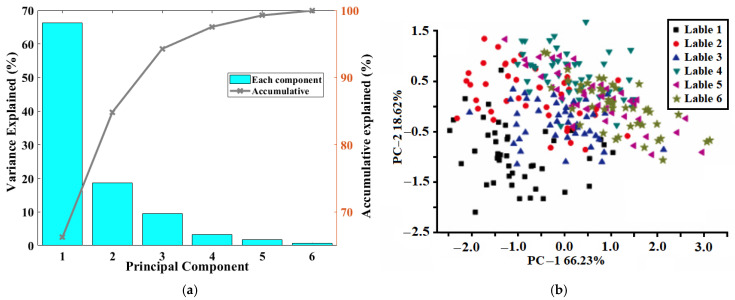
Explanation of samples by PCA. (**a**) The explained rates pf top PCs. (**b**) Scatter of samples projected by PCA.

After the background signals of the Raman spectrum have been effectively removed, those left are discontinuous spectral signals that are then amenable to PCA. Figure 3a illustrates the contribution of the initial principal components and the cumulative percentage of variance explained by the original information. It is observed that the first principal component alone accounts for 66.23% of the variance, with the top three principal components collectively contributing to over 92% totally. From the two-dimensional principal component scatter plot in Figure 3b, it is evident that samples from all categories are clustered together, with significant overlap occurring between adjacent categories. As the category label number increases, reflecting the aging duration of the transformer oil, each class center is sequentially arranged from the lower left corner to the upper right corner along a nearly 45° direction. This pattern suggests a correlation between the aging time of the transformer oil and its spectral characteristics, with longer aging times associated with samples distributed more towards the upper right corner. However, despite the potential relationship between the aging time of transformer oil and its Raman spectral features, the extensive overlap between samples and the inability of the unsupervised principal component analysis method to effectively differentiate them indicate the need for a more robust approach. Consequently, this study will integrate a supervised support vector machine (SVM) classifier with PCs to discern sample categories.

### 3.4. Classification by SVM on the Reduced Spectra

When applying SVM for spectral classification, the reduced-dimensionality data (by threshold cut or PCA extraction) are more conducive to finding optimal decision boundaries with clear margins. SVM’s strength lies in its ability to handle high-dimensional spaces and nonlinear relationships through the use of kernel functions, which allows it to effectively classify data even when they are not linearly separable in the original feature space, as Figure 3b shows. Here are two key parameters that determine the boundary of supported vectors (i.e., features of samples): the penalty coefficient ***c*** and the kernel function parameter ***g***. In this work, three search methods are used to find the suitable pair of these two parameters in the default boundary ranges [30,32], and they are grid search (GS), particle swarm optimization (PSO), and genetic algorithm (GA), respectively. Moreover, due to the limitations of sample numbers, a cross-validation set in the training set is used instead of an external validation set, and five-fold with the ‘syst123’ selecting method was set in the training process [30,32].

**Figure 4:**
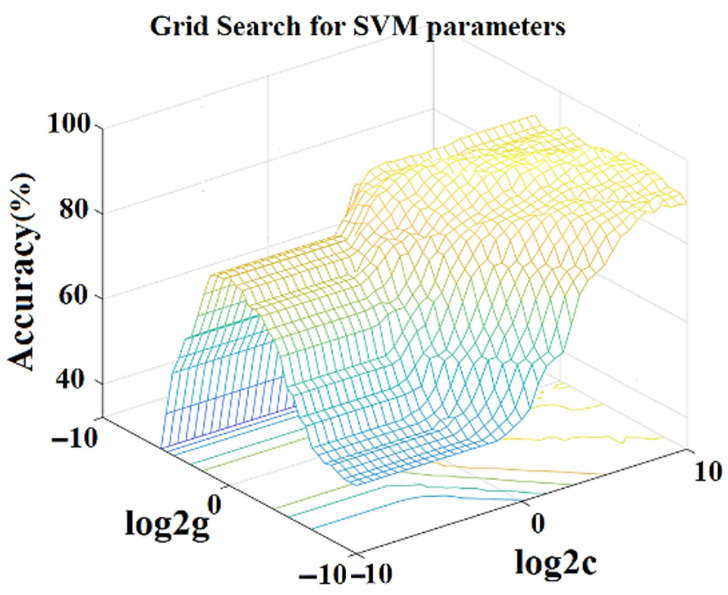
The key parameters of SVM classifier optimized by grid search method (color for different accuracy).

The cropped spectra by intensity-threshold reduction were used as the input of SVM classification. The three methods were orderly used to optimize SVM’s parameters to develop the SVM classifiers. The grid method is to search the optimal parameters in a given parameter space; divide the parameter space into grids with the same length, wherein each grid point represents a group of parameters; bring all grid points into the SVM to verify the classification effect; and enumerate the optimal parameters by force. The search result is shown in Figure 4. PSO is to find the optimal solution through cooperation and information sharing among the individuals in the population; GA generates multiple starting points randomly and starts searching at the same time in the process of optimization, and the search direction is guided by the fitness function, which can find the global optimal solution in the complex search space. Table 2 shows the results of SVM classifiers to be optimized by different methods and their performances on the oil aging samples.

Compared to the grid search method, both PSO and GA yielded identical cross-validation accuracies of 93.75%, slightly worse than that of the GS-assisted SVM, which obtained 94.58% accuracy in the cross-validation stage, where just 13 samples were misclassified. However, the optimization times for PSO and GA were longer and more than 1200 s in the same computation device conditions. This is attributed to the convergence mechanism in PSO, where particles achieve convergence along trajectories, and their velocities are inherently limited, resulting in a longer time searching the entire solution space. For GA, a single encoding scheme cannot fully represent the constraints of the optimization problem, necessitating the use of penalty functions to transform constrained problems into unconstrained ones, thereby significantly increasing the workload and solution time. According to Table 2, the accuracy of SVM by the GS optimization method achieves the best performance on the prediction set and reaches 90.28%. The prediction set comprises 144 samples from six categories, with 14 samples misclassified by the GS-assisted SVM. Therefore, the grid search method is selected as the most effective optimization approach.

### 3.5. Classification by PCA-SVM

**Figure 5:**
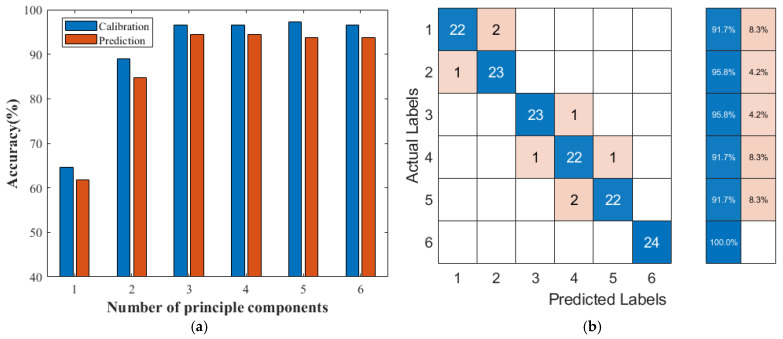
The performances of SVM classifiers optimized by grid search method. (**a**) Performances of SVM with different numbers of PCs. (**b**) The confusion matrix of the best SVM classifier.

The compressed principal components extracted by PCA were used as the input of SVM classifiers, and the number of PCs increased from one to six, where they represented over 99% original information, as Figure 3a shows. In this section, the key parameters in SVM classifiers were optimized by the GS method, which was proven to be the most efficient in the above process. The optimized SVM classifiers with different numbers of PCs and their performances are presented in Figure 5a, and the classification accuracies for calibration and prediction sets varied greatly with different numbers of PCs. With increasing the number of PCs involved, SVM classifiers performed from the worse to the better. When the number of PCs reached five, SVM classifiers achieved the highest classification accuracies, with 97.01% in the calibration set, but the accuracy in the prediction set was not the best and slightly lower than the classifier with three PCs, where the SVM classifier obtained the best performance of 94.44% in the prediction set, as shown in Figure 5b.

With spectral projection by PCA mapping, SVM classifiers can achieve better performances than that without spectral compression in total. After the amount of input dimensions reduction from the spectral threshold cut to the PCA projection, the classification accuracy of the SVM classifier increased from 90.28% to 94.44%. The contrast between long aging and short aging is clear. The actual category of the samples in category 5 and 6 is exactly the same as the predicted category, and the samples in categories 1, 2, 3, and 4 are misclassified by eight samples in total, as shown in Figure 5b. The effect of distinguishing different aging times in a short time is not ideal. When the actual aging category is one label, two samples are classified into category 2, and one sample from category 2 is misclassified into category 1. With increasing the aging time of oil-barrier insulation, four samples were misclassified in categories 3, 4, and 5. With increasing the aging time of transformer oil, there may be some inaccuracies in the sample classification by the SVM classifier. A small number of samples may be misclassified, yet these misclassifications are likely to occur within adjacent categories rather than more distant ones. These misclassifying results mainly occur between adjacent categories, and these have also appeared in other studies [8,11,14]. A further subdivision mode can try to solve the classification error between adjacent samples. This characteristic effectively minimizes the risk of significant misdiagnosis of transformer oil conditions, thereby ensuring the normal operation of the transformer.

## 4. Conclusions

This study employs indoor accelerated aging experiments to simulate the degradation of oil-immersed barrier insulation within transformers. Raman spectroscopy is utilized to examine samples at various stages of aging, coupled with spectral analysis techniques and pattern recognition to facilitate a rapid assessment of the transformer oil’s condition. The raw Raman spectral data undergo S-G smoothing, ArPLS baseline correction, and the threshold removal of Raman low signals. The model parameters for SVM are optimized using grid search, PSO, and GA, and with grid search it demonstrated the shortest runtime while maintaining excellent optimization performance. Following intensity threshold cutoff and PCA for dimensions reduction, the classification accuracy of the SVM model increases from 90.28% to 94.44% in the prediction set. The results indicate that the integration of Raman spectroscopy with machine learning methods can effectively evaluate the aging state of transformer oil, ensuring the healthy operation of transformers and eliminating potential safety hazards.

## Figures and Tables

**Table 1:** The different timespans of aging process and sample’s division.

Aging Timespan	0 h	24 h	72 h	120 h	168 h	336 h	Number of Samples
Labeled class	1	2	3	4	5	6	384
Calibration set	40	40	40	40	40	40	240
Prediction set	24	24	24	24	24	24	144

**Table 2:** Comparison of different optimized methods for support vector machine (SVM) parameters.

Optimization Methods	Runtime	Accuracy of Cross-Validation	Accuracy of Prediction
Grid search (GS)	937 s	94.58%	90.28%
Particle swarm optimization (PSO)	1243 s	93.75%	88.19%
Genetic algorithm (GA)	1576 s	93.75%	88.19%

## Data Availability

Data will be available on request.
